# Dr. William Asa Wheeler

**Published:** 1912-03

**Authors:** 


DR. WILLIAM ASA WHEELER, died at his home in Portland, Me., Jan. 20, aged 58. He was a graduate of the Medical School of Maine, 1876 and of P. & S. 1877. For some years he was an officer of the U. S. Marine Hospital Service. He was formerly Professor of Surgery in Niagara University, and later, President of the Portland Board of Trade.

